# Supplementary material for: PtrABR1 Increases Tolerance to Drought Stress by Enhancing Lateral Root Formation in Populus trichocarpa
Source: Int J Mol Sci. 2023 Sep 6;24(18):13748. doi: 10.3390/ijms241813748 (PMC10530772; doi:10.3390/ijms241813748)

Figure S1 The promoter sequence of PtrABR1. Start codons in red.

ATCCCGCTGGCATACAAACTATTATATTTCTAAACAATTAAGATCCTTTTCAGATCATGATTAAA
TTCGATTGAAAATAGAAAGAAATATCAATTTCATATCTTTTCGACTAAACAATCTAAATCTTATC
GGTAAGAACACACTTGACACGAGACGGGTCGCGGCACAAACAACTATAGCCACACAAGAGG
ATTAAAATTAAAGTCTTAATCGAAGCTGGTCTAGTGGAGATAGATGAAGCACCCTTTATAATTA
TCTTCCAATCTTTGATCGTCACCGTTTATACTAGAGATAAATTTCAATTTATTTATACCAACCTTC
GTCGAAGGATAAACCTTCTCGAGCATAGTCACTGTCGTAATCAAAAGTAATTGTCTCGTGGGCC
TCGCACAAAACTAGCCGAGATATGAACGTACTATAAAGCATTTATATTTTTTAAGACTACTATA
CCTAATATTGACACATATACACACATGTATAATATTCCTAATCTCTCCATTTAATACACACACCC
TCTTTAATTGTATATCTAATGAATAATTTATAAATGTTTAAATGTTTTATTCGATAAATAATTGCC
AAGATACGAACGTAAGAATCTAATTCATGAATATCTATTAACATTCCTAATTTGAACTCACCGA
AGCCAGATTAGCAACTAAGCTTCCAAAACAGGAGGTTAGTTTAGCTACACCTTGCCTGTTCGTG
GTTAAACACCAAAAGATCATGAATCTTGATGATCAGCCTCTGCATAATTATAGAGTAATTAGGC
ATGGTAAATTACCAGAACCGGGCCAGTCAATTTTTTGACCAAGTTGAAGCAGAGAATTTCATCT
GTTCTTCTGCGTAAATGACTTTAATTGGATAGTTTCGTTTTTATTTTAGGATTTCAGTAAATAATA
GTATTGGCAATTCCACACAGCTGATTGTCAGAGGTCAACCGAGAGACGTGTTTTGACATTACAT
ATCATTTAGAAACAAATATGTAAATACTTGGCCATTAAGAAAACCAACTCAATATTTTACTTCA
TTTCATCTGCTCGCAAGAGTTAAAACTCGGAACTTCTTATCCATGGAAGGTTCAGTGTTACAAA
ATTGTACAAGCAAGGGGTTTTAATATAGAGCATTGACCCCAGATATTATATTATATAAAACAGT
ATCGAAGGTTGTGGTCCAATGGAACTAACAGAAGAGTACAATTTGTCTACAATCCAATGACCTC
AGTTTTACTCGGTGTATGCTTTTGTGTTGAACAAAATGAAAAGTCAGAACAAAAGCAATTATTT
ACACAATCCCGCTCAAAACTTCAACCACGTTCACAGGGACATCTCTCAACTTTTACAGCTCAGG
AAAATGCTGGAAACAACTTAAGAGCCATCATCTTCTAGCCCAAGTTGCTGAAAACCTTTCGACA
GCCCACCTAGCTAATAATTATGGGCTATTCTTAAATTAATCTGAAGGCCCGATCTTAAATTAATC
AGCTTTCTTGTCGGTTATGCTTGTAGGAACTCAATTTGGACACGCTTTCTGTTTCTTATCTAAACT
CCTACTTGTTCTAACTTGAAATGGCTTTCCCCTGGGGTTCCATAGAACCTCAAATTTATCAGTCA
AATCTTATGTGTGAACGGATATTAAATTCGGGATTCTGTACTTAATTTTTTTGTATCCCACCTCTT
AAAGGGAAAAAAAAAAAGTTAAAAAATTCACAAGCCAAAAACCTTTTTACATAAACCTCTCCCA
GAAAACATGTATCTCCGTATTTATCACAAAGGTGGTGCTAGCTCCTGGTTCAGCGAATGATGCA
GAAATATTCTAGCACTATTTCAGTGACGGCTGAACAAGTTTGAATTCTCTGCTTCATATATAACC
CCCTGTCTTCACTATAACTTCAAAAAAAAGGTTCTCATACAATTTGACAAAAAAGAAAAAATCC
AAAAGACCCGGCTTGATATCTGTACCCAGCTTTTTCGTTCGAGTTACATG

Figure S2 Characterization of *PtrABR1* promoter transgenic plants. (A) PCR confirmation of *PtrABR1* promoter transgenic lines. M, DNA marker. The DNA marker is 5000bp. (B) GUS expression analysis of transgenic plants. Student's *t*-tests, ** ($p < 0.01$), and *** ($p < 0.001$).

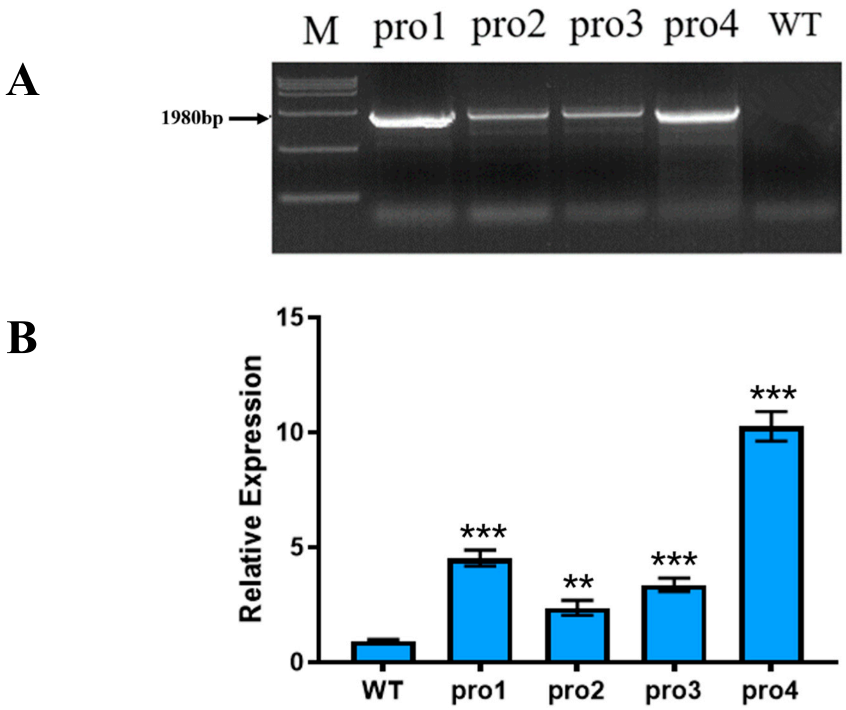

Figure S3 Characterization of *PtrABR1* transgenic plants. (A) PCR confirmation of *PtrABR1* transgenic lines. M, DNA marker. The DNA marker is 2000bp. (B) RT-qPCR analysis of *PtrABR1* transgenic plants. Student's *t*-tests, *** ($p < 0.001$).

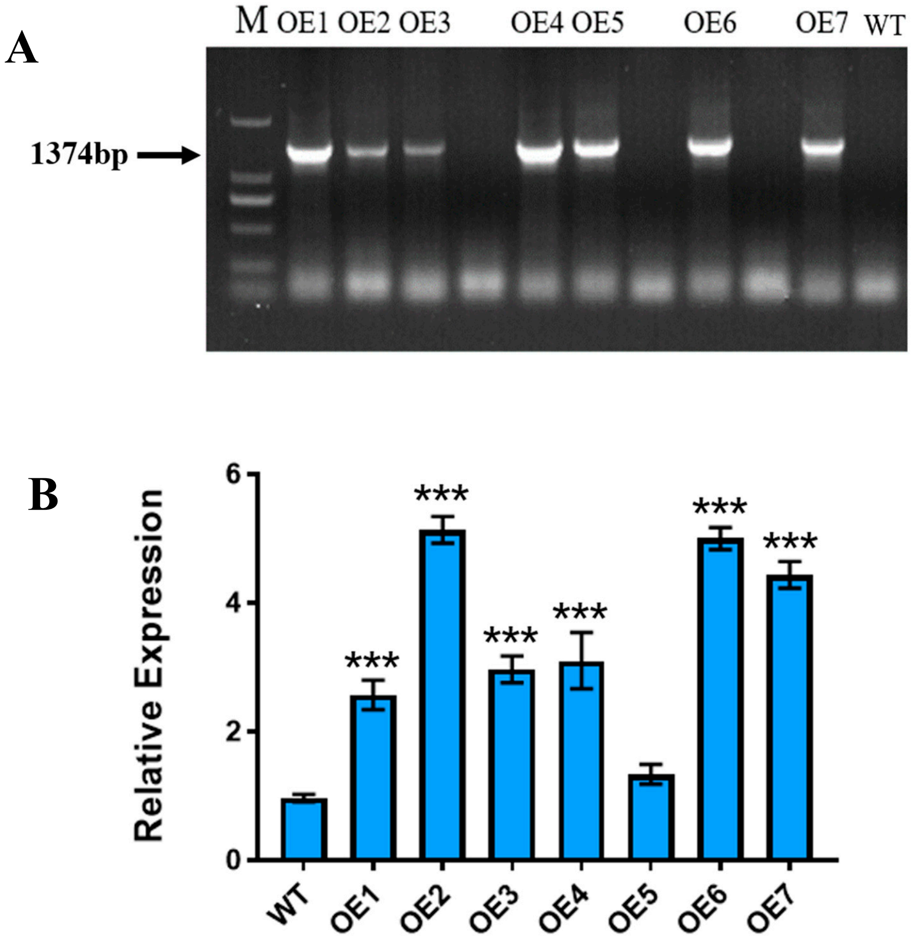

Figure S4 The phenotypes of the aboveground part of *PtrABR1-OE* and WT plants treated with 6% PEG6000. Bar, 1 cm.

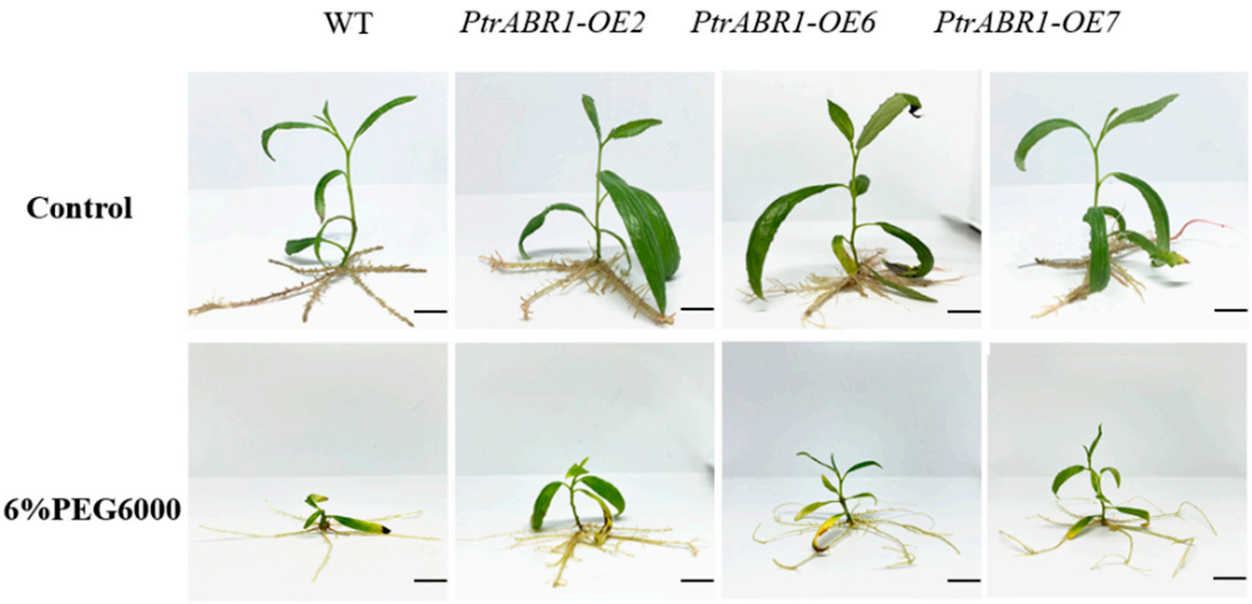

Figure S5 *PtrABR1* putative CDS sequences of upstream genes.

(A) The CDS sequence of *PtrYY1*

ATGGAGGCTCATCATCATCATCATCAGTTCACTCACAATATATTTGAGAGACGCCCCATTATCA
GATCAAAGACTCCTGCTGTTAAATGGTTCAAAGAATGGGTGCCTCAAGATGTGGTGGCAACTGG
TGGAAGGTGTTCTCTTTTCAAATGGGTAACAGAAAACCAATTAAATGCCTTGAAAGAGAAGGC
AAAAGAGCCCCAAGCACCAGAGCCAGAACCAGAGCCTACTACTGAGGTTCTTTTCCTTTGCAGT
TTTGAAGGGTGTGGTAAGACCTTTATCGATGCTGGTGCTTTGAGAAAGCATTCTCATATACATGG
AGAGAGACAATATGTTTGTCACTATGAGGGATGCGGAAAGAAATTTTTGGATAGTTCCAAGTTG
AAAAGACACTTTTTAATTCATACAGGGGAAAGAGATTTCATATGTCCTCATGAAGGCTGTGGTA
AGGCATTCTCCTTGGATTTCAACCTGAGATCTCACATGAAAACACATTCACAAGAAAACTATCA
TATCTGTCCATACCCAGAATGTGGAAAGAGATATGCTCATGAATACAAGCTGAAGAACCACAT
TGGTTCTCATCATGAAAAGAATCCGACTCCAGAAGTGGTGAAATATGCTACACCTCCAGAGAG
GATACCTAAGAATGCCAAGACTCCTCCAGGGGTTTATGGCTCTGCATCATCAGATCGACCATAC
ATTTGCCCTTATGAAGGGTGTGAAAAGGATTACATCCATGAATACAAGCTCAAACTCCATCTGA
GAAGAGAGCATCCTGGCCACATGGCAGATGAGAATGCTGAGAATGCAACACCTAATGCTGACA
ATGAATTGGATGAAGCCAGTGATCAAGATGCTTATGGTGGAAAGCGTGTGAATGGGAAAAGTC
AGAAACAGAGCAGGTCCAAGCCAAACTTGAAGATGCCTCCTGTAAAAATCAGACAACGGAAA
GGCTCAAGTCCATCACCTGCCACATTGAATGTAGTGAAAAAACCTTGGACAATTAAGGATGAG
ACTTATGAAGAAGAAGAAGACAGTGAAGAAACCGAGGAAGAGGATCGTGACAATGCAGAGG
ATGGATGGAGGTATGGAGGAAACGAGGATGATGATGAAGAGACGGAGGATGAAGACTAG

(B) The CDS sequence of *PtrSPL10*

ATGGAGTGGAATGGCAAACCCCACTTACAGTGGGACTGGGAGAGCCTGATAATGTTCAATGGA
ATAACAACTGAAAATTCTAAGCAGTTAAGCCCAACAGATTTGGAAACTGATGGAGAAAAAGG
AACCGACTCTGGGTTTTTCTATTCATCTGGGAGTGCAAGCAGAAGCGGTGGTTCTAGCTCTGATT
TGGAACTTGCTTCTTTCTCAAAGTGCTCGAAGTCAGCTTCCATCAATTCTTCATCAGCTGGGGAA
GTTAAAACATCCAAATTCACTTTGGAGGCCTCTAAAGCAAATCCATCTGATTACAATAAGAAAG
AAATTGGAAAGGCTAAGACAGCTAGTATGTCTTCCACAATTGAGGCTGCAGGTGGTTCAGGTG
ACCAGCTGCTTGGTTTGAAGCTTGGTAAACGAATATACTTTGAAGATGCTTGTGCTGGCAACAA
TGTTCAGTCGTCATCATTTTCTACAGTTCCTGTGCCCTCTTTTACTTCAGCAAAGAAATTGAAGTC
CACTATTCAGAGTCAGCGTGCTCCATGCTGTCAAGTGGAAGGCTGTAACCTTGACCTCTCATCA
GCTAAAGATTATCATCGCAAACATAGAGTTTGTGAAAGCCATTCAAAGTGCCAGAAGGTCATT
GTAGCTGGTTTGGAACGCAGGTTTTGCCAGCAGTGTAGCAGGTTCCATGGCCTGTCAGAGTTCG
ATGAAAAGAAGAAAAGCTGTCGCAGGCGACTTTCTGATCACAATGCAAGACGCCGCAAACAA
CCAGGATCGGTCCATTTAAATTCAAGAGTGTCTTCATCATTATATGATGAAAGGCAACAGATGA
GTCTTGCTTGGGACAGGGCACCACTTGTTCATGCCAGGCCTAATGCAAATTTGACGTGGGAAGG
CACATACATCTCCAAGTTCACAATAACAAAAGATTATATAGCAAAGCCTGCAGAAATAGGTGG
TAATGATGGGCAGTTTCACTTGCCTGGCTTTGATCTGACAAATGGCATTGATATTCAGCACCATC
ATAAGTCTAATAGCTCCTTACCATCTAAAGGTAAGGGCACTGCAGCTGAGATTCTCAACCAAGG
TTTGGAAGAATACATCATTCCTTCCAAAGCGGAAGCAGCACCAGAATCTCATCGTGCTCTCTCT

CTTCTGTCAAACAATTCATGGGGTTCACGTGAGCCGCAATCTATTTCATTTGAACAACCCGTGCA
TACAAATCACACCACTCAGTCTGTGCTGCAAGTTATACCCCAAAACTCACCACTTGCTTCATCA
GAGTATTGGAGGACTGAGCAACCGTCAACTGACTCTCAAGTGCATACCTTGACGTCTCACTGA

(C) The CDS sequence of *PtrABI4*

ATGCGCACCAGTGCTGCCTGTTTACTCCAGTATGAAGCAGCAACTTTCTCTCTCTCCGAATACAA
AAATATAAATACATGCAGTCATTGTAGATCACCGCCCTCATCCGCCATTTACACACATGACATT
CAAACCAGCGCACAGGCAAAGGCTACTCTATACTTCGACAGATCAATAACCAAAGAACGAGG
CCCCCTCAGTCTTAATAAAGCCTTAAAAGCGATTCACTTTATAATGGACAATTCATCTCTCTCTC
ACCCTCCCCAAGAACCCACCACCACCACCACCAAATTATCATCCAATGAAAAAAGCACCGATA
ACAATACCACCGCAACCACCCCCACTACCGCCACAACAAGTGACACAAACAGTAACAACAAC
AGCAGTGGCAATAGCAGGAAGTGCAAGGGCAAAGGAGGACCAGACAACGGTAAATTTAGATA
CAGAGGAGTTAGGCAAAGAAGCTGGGGCAAATGGGTAGCAGAGATCCGTGAGCCAAGAAAAC
GAACCCGTAAGTGGCTTGGAACTTTCGCCACCGCAGAGGACGCAGCACGAGCCTATGATCGAG
CAGCCTTCATCCTTTATGGCTCCAGGGCTCATCTCAATTTGCAACCCTCAGGTTCCTCTTCCTCTG
CTCAGTCCGGATCAACTTCTCGCAACTCTACCTCTTCCTCGAGCCAGACTCTTCGTCCTTTGCTCC
CTCGTCCCCCTGGGTTTGGTTGTGGCTTTGGTTTCACTTTCTCTCTCTCAAATCCAATGGCTTCTCC
GTCTGTCACGGCAGCTTCATCGGGATTTACTCCATACGGGGTTAATTGTTATTCGAATAATGTTG
TTGGGTCGGCCTTACAATGTTCTAGTACTAATGAAATGCCAGGGCAAAATCACCAGCAAGTTAT
GTTACAAGGCTATCTCATTCAACATGGGGCTAATACAACCAACCCCAATAATATATTTGTTAGT
TCTAGTGTAGATCCATCAACAACAACCTCGTATCAAAATCATTGTCATCGTCTGCCGCAGCATC
ATGCGTACGATGATGTTAATGCGTTGGGGGGTTCCGTCGGGTCGAGTTTCTCTCTGTCTGGCAGC
AATACTCCTCCTGTTGTTGCACCAGCGGGTCATCTTCTGCAGGATCCGGTAATGCATATTGGACC
TGGATCTCCATCTGCGTGGAATGATGAGGAGTACCCACCGCCTAGTATTTGGGACGATGAGGAC
CCTTTCTTGTTTGATTTTTGA

Figure S6 Acquisition and characterization of *PtrYY1* transgenic plants. (A) Infiltration of *PtrYY1* transgenic stem segments and screening of resistant shoots. (B) PCR confirmation of *PtrYY1* transgenic lines. M, DNA marker. The DNA marker is 2000bp. (C) RT-qPCR analysis of *PtrYY1-OE* lines. Student's *t*-tests, ** ($p < 0.01$), and *** ($p < 0.001$).

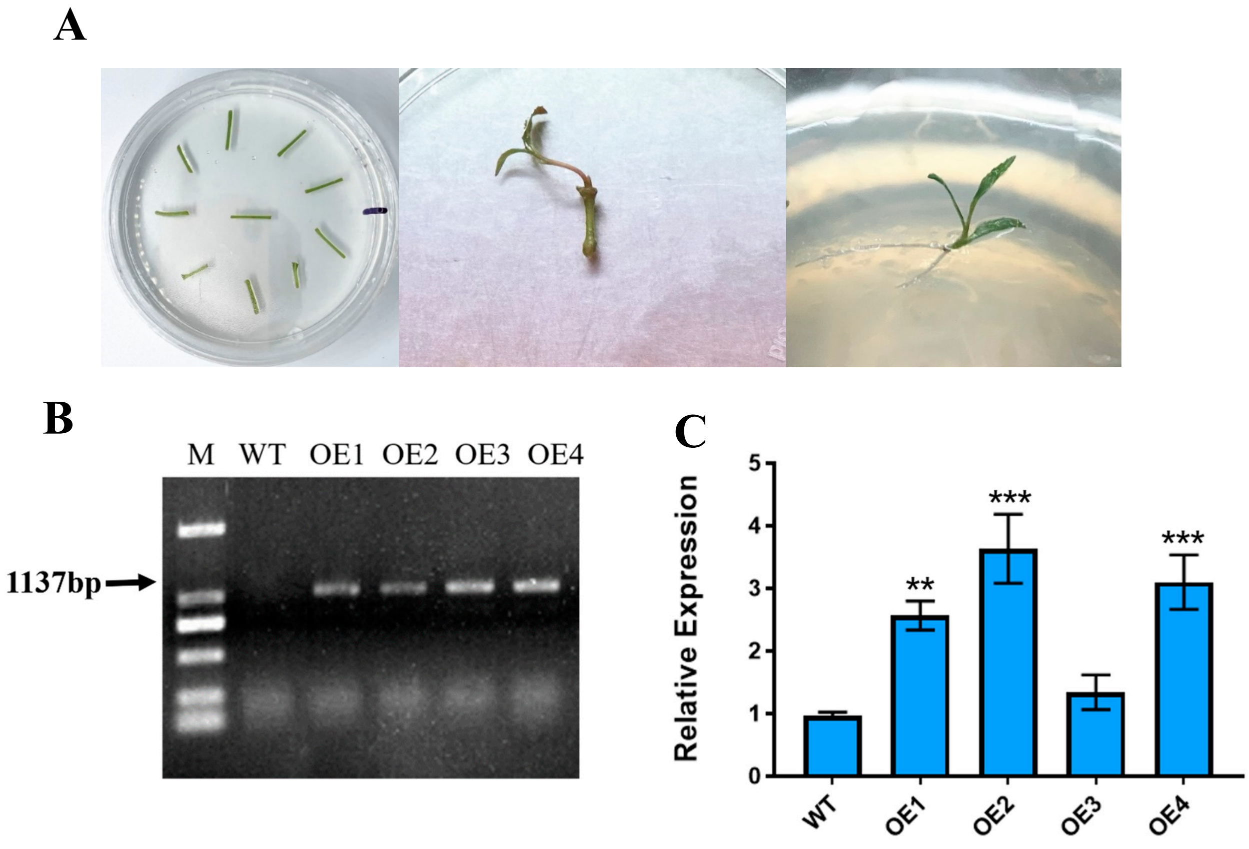

Figure S7 The phenotypes of the aboveground part of *PtrYY1-OE* and WT plants treated with 6% PEG6000. Bar, 1 cm.

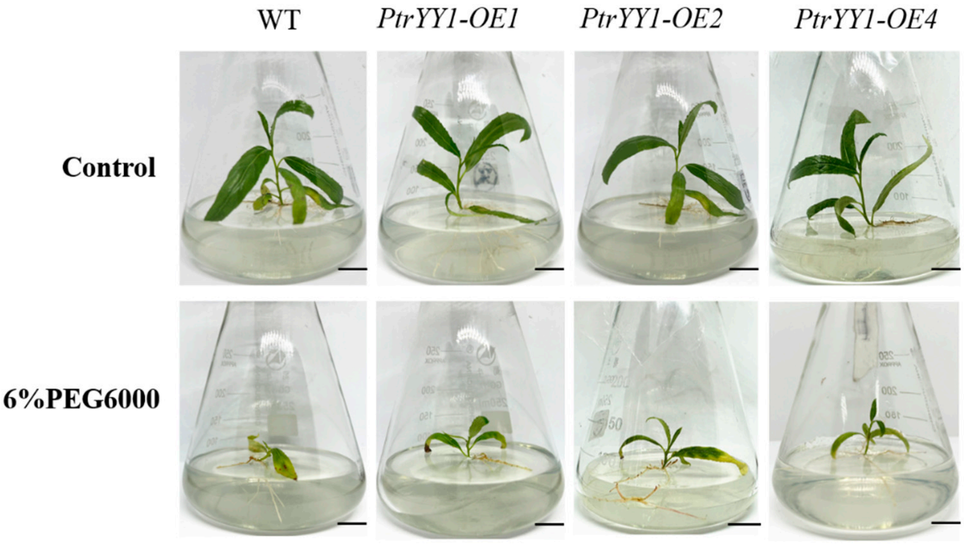

Figure S8 Promoter sequences of potential downstream target genes. Start codons in red.

(A) The sequence of the *PtrGH3.6* promoter.

ATAGATGTTGACGATGCATCGAAGGTCCAACGACTGCAGGCACCACATAGAGAGAAAGAGGA
GGCCGAGACAAATCAAATAACTTGATGGAATAATATTTTTTACCACCAGAGTTAGCTGCATGCG
CCGCCCAAAGGTAGGTGATGCCTACAACACATGAGTCAACAACGTTTGACATTTTTTTAAATAA
TACTCTAGCCTTAAAACCTAAGCATTATTAAAAAAAAAACATTTTGAAATTACAAATTAGCCCTT
CAAGTCAAGCTTTTGAAAATTTGAATCCAATGGTAATAGAGTAATTATATCGTAGCAAAAAATA
TGAAATGCCTAAAAAGATACTAGATCATAGCTAAATGATTATTTGTTTTTAAAGGTAACAAAGT
CATTTTACTGTGAAAAAAATTAAAAAAAAAACAAGACATCCCTTAACAATTCTGCTTGAATTGA
TAATAAGCAGAGATGGATCAAAAGATCATACAATAATGTTGTTTGACTCTAAGAAATAGGTTTT
TCAGGTCAACACCCCTATTAAATCGGGTGTTGTGAAAGAAGTCAGATGGGTAGTTAGATTGAAT
GATAAAATATGCACATTAAAATGGTTTGATCGTTTTGGCTATTGTCCAATTATTTTATCAAATAT
CCAATTATTTTTTAAAACAATCTCTCTAAAAATATCACAACTCAGAAACACGACATGTATTAAA
TGTCAAGTTTTTGAATCGTGATATTATTAAAATATTATTTTTCTGAATCATGATATTTTTAAACTA
TATTATTTTACTGAAATTTTTTTATCCTCTGATATTTTAGCATTTAATTATATTTTAGGTTTTAACTG
CACACTTTATCCAAAATATAATGTAAAGTTAGCATTGATGTTTGTATAATGCAACGGTCAACCT
GCGGAACGTAGTCAAACCTGGTTTCACAAATTCTCCCGGTCTCTCTGCCCATTTAACAGTTGTTT
GGTCTGGTGTGCTGTAAGAGATGATGACATACGATGAACTTGATCGGAACCTTGAGGACTTGAC
CAAGAACGCCGCACATCACCAGCTCCAGACCCTCCACTCCATCCTCCAACACCAAGCTAGCGT
TGGCTGTTGGGTTGGTTGCCATTAATGTGCAACAACCATTGACAATTGCAGCAGCTGCAGCTGG
AGTTGCACCAACCATGTGCACCCATCCAAGATGTCAAAGGTTGGCAGCCACCATTGTTGAAGA
AGAGCTGGAGTTGGCAGCCACCTTTGTTGAAGAAGAGTTGGAGCTGGTGGCACCATTCTTGCCT
ATAAATAGGCAGCAAGAGAGTAGAGCAAACTGGGAGAAAGAGAGGTGTGAGAATGTGAAGA
GTATAGAGAGAAAGTGGAGAGCTGCAATGGCAGCAGCCTTGTTGCCATTGCAGCAGCTGCAAG
TGCAGCAATGAGAGCTGGGAGTAGAGTTTGAGTGAAGTGATCCTCCTCCTCCATGTATATCCTTT
CTCTAATCTCAAATAAAATGGACTCTCTCCCGTGGATGTAGGCGGTTTTGCCGAACCACGTAAA
ATATTGTGTCAGTGTACTTTACCCTCCTATGAGCAAATATCAGTACACCCCCGGTCCGCGCAAG
GGGAGCCGGAACAAGCTAGCGTTGGCTATCTTCAGCCTTACCTCTCTGCCTGTCATGCCCCCGTC
GATGCCGCTACTTTTCGAAGCCAGGTCCCTTTGTCTTCCTACGATGACTATTTTCATCTCATCAAT
CAATTGGCCAATGGAGACATTGATCACCACCAGCCCCTCCTCTCCGCTGATCCTCTCCTTTGTTT
CTTCTACAGGTACTGCTTTGATTTCTTCTTCAAGCCCAATAGCAAAAAAAAAAAAAAAAAAATCG
AGGAGGAGAAGAAAAAACCTATCTTGTATCAGATTATAATTTATGTTTTGATTTTGTTGCTCTGG
TACCAGTACAATGAAGCCTATCTTGTATCAGATTATAATTTATATTTTAATTTTGTAGCTCTGGCA
CTAGTACAATG

(B) The sequence of the *PtrPP2C44* promoter.

CTTTTTATTTACCCTAATTCATAAAAATATAATGAATATCAATCCTAATAAAGTTTAATGAAGAT
TTCTCCAAATATATACGGACATGAAAAAAATATTATTGTTGGTCAAGTATTTAGAAGTTGTTTGG

AATTGTGGTGTATAAGTATTTTTTTAAATAGTTTTTCACTTAAAAATATATTAAAATATTTTTTTTT
TATTTTTTAATTTTTAACATTATTACGTTGAAATTATAAAAAAAAATACTAAAATAATATTAATTT
AATACTTTTTAAATCAAAATTACTTCTAAAAAATATCTAAAAACGTAATACCCCTTGTTCTGACC
GTCAAAGACGATCGCCGGCAAGCATTGGCACGAATTGCCTTGTTTTACCGATAGATTAGGAAAT
AGAACAGGCATGAACCAATGCGTCTAATTTAATAGTGTAATGATGATGACAATGTACCAGTTCA
CTGTTCACCTTTCTTAGTTAATGTGCCTGAAATTATGGCCGGGAAGAACAAAGCTGGCCGTGGC
TCAAATTTACATGACTGACTCACCAAAAAAGCATAGAGAGAGTCATTAAAAAGATTGAGTACT
TTTTTTTTTTGTTTTTAGAAAGTAAATTCTAGAAAAGTGAATTATTTTTTGATGTTGGTTATTGTAA
TAGAAAATAAGTTGAAAAATATTTTTCAGTGTTTGGTTATGTCATGGAAAATGAGCTGGAAAAT
AACTTATTAATGTTTTATTTTTTTCAAATTTATTAAAATAATAAGGAACAAATCTTACAAATTAA
AAAGTTGAATGAGAATGAAATTGAAAAAAAATATAATTTCATAAATTATCTCAAATAAAATAA
ATAATAATCAAAATAATAGAGATCAAATCTAAAAAATAAAAAAATTAAAAGATGAAGAAATT
AAAATAATAATAATTAACATTTCATAAATTATTTCAAATAAAATAAGTAACAATCAAAAGAAT
GAAAACTAAATTTGATAGATAAAAAATTTCAATTAAAAAATGATAAGGGAAAAACAAATAAC
AATTATAAAAATGAGGACCACAGTTAATATAAAAATTAAATTCTAATGGATGAAATTGAAAAA
CAAATATTTAAAATAAAATATATATATATAGCAATCAAAAGTTTGAGGATCAAATTTGATATAA
TCAACAAATAATTCACCTTTTTTATTGGAAAGTGTTTTCTGTTGACCGGGAAAACTATTTTTCGAT
AAACAAACACAGCTTTAGTTGAAAACATGCATGACTAACCCTTGTCAAGTCTTCGCGCGGGGG
GTAAGGTTGTTTGTCGCCATTTCCTTCATCACATACTTAATATTTTAAAACGAGATTGTTTTAGTT
TTTTTTAATAAAAAAAGTTATTCGAACAAAATAAATTAAAAATTTAACATAATGTTATTTTCAAT
AAGTTTTATTTTTTAAAAAAACTTTAAACCAATTCGGTCATGTATCACATGAGTTTTTGATCGTAT
TAGCTAAATTATTGAATTTTAACTAGGTTAATAGTTTGAAAGTAAATCTAGCTCGAAATAAGTTT
TTAATCAGAGAGTGCCTAACAAATTTGATGACCATTTATGATAAAAGTGAAGAAGAGTCACTC
ACACAGGTGACTATTATTATGTGTTTAACATGGCATTTAACGAAAATGGCAGAAGGTAGAAAC
CGACTGTAATAAGGCTTAATTTTCTTCAACAGAAAAAATAAAAACCCATAATTTTCCCATTTAA
TACACAGTGACCTAAAAATAGCACTTCTTGATAATAGTCTTTTACACCTTTAGACCATAGATTGA
CTTCAAACTTCTCTTTTGATTCATTTTCAAAGCAAACACCAAAGCTTTCTGCAAAGCACGACTGT
CTCCTTCCCTTCCTCCACCCCTCTCTCCAACCCTCCTTCCCTCTTTCTTTTAATTTTCCAAAACCAT
TCCCTTTTGATTTGCTTATAGTATACGTATATATATCTTGGCCTTCAAATACTACGTATTCAAGGG
TATG

Figure S9 *GH3.6* and *PP2C44* 2000 bp in front of ATG for cis-element analysis. Different colours represent different cis-elements.

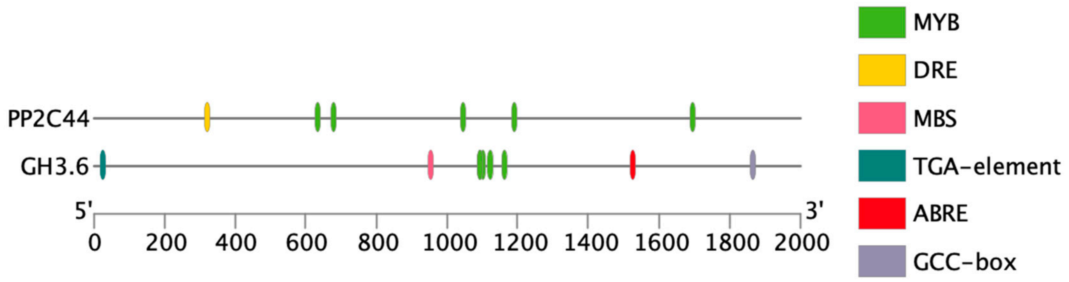

Supplement: Supplementary file 1 [file ijms-24-13748-s001.zip › ijms-2582007-supplementary/Supplementary materials.pdf]
